# The Transactions of the American Medical Association

**Published:** 1852-01

**Authors:** 


*The Transactions of the American Medical Association.*—Vol. IV., pp. 677.

This volume is the fourth one issued by the American Medical Association, and as a whole, is superior to either of its predecessors. The reports are all prepared with care, but we greatly prefer the divisibility of labor adopted at the last meeting of the association, which cannot fail to elicit a larger amount of valuable information, than could have resulted from the former organization of the committees. The chief defect in most of the reports of the committees of the association, is to be found in the vastness of the field occupied, and the comparatively slender tillage bestowed upon any particular part of it. The committees were not so much to blame for this defect, as the organization under which their reports were made. Generalities were absolutely necessary, and the limited nature of the reports precluded a careful and minute analysis of these generalities. The twenty-seven committees appointed to investigate particular subjects, are free from this objection, and are not expected to embrace a comprehensive field of inquiry. But while their investigations are necessarily limited, it is to be hoped that they will be thorough.

The association has assumed a position, which, so far as human foresight can penetrate, is likely to ensure to it a long career of usefulness. Without high sounding pretensions, or vain boasts, it has already manifested by its works, its great capacity for the advancement of the best interests of the medical profession. We may have occasion to refer again to this volume, and in the meantime, we would recommend it to our readers as well worthy of their perusal. The prize essay, by Dr. Dalton, is highly creditable to the author as well as to the association.

